# Pre-Emption of Affliction Severity Using HRV Measurements from a Smart Wearable; Case-Study on SARS-Cov-2 Symptoms

**DOI:** 10.3390/s20247068

**Published:** 2020-12-10

**Authors:** Gatha Tanwar, Ritu Chauhan, Madhusudan Singh, Dhananjay Singh

**Affiliations:** 1 Amity Institute of Information Technology, Amity University, Noida 201313, India; gatha.tanwar@gmail.com; 2 Center for Computational Biology and Bioinformatics, Amity University, Noida 201313, India; rchauhan@amity.edu; 3 Endicott College of International Studies, Woosong University, Daejeon 34606, Korea; 4 Department of Electronics Engineering, Hankuk University of Foreign Studies Seoul, Yongin 17035, Korea

**Keywords:** smart wearable, smart health, heart rate variability, onset detection, hidden markov model, SARS-Cov-2, Covid19

## Abstract

Smart wristbands and watches have become an important accessory to fitness, but their application to healthcare is still in a fledgling state. Their long-term wear facilitates extensive data collection and evolving sensitivity of smart wristbands allows them to read various body vitals. In this paper, we hypothesized the use of heart rate variability (HRV) measurements to drive an algorithm that can pre-empt the onset or worsening of an affliction. Due to its significance during the time of the study, SARS-Cov-2 was taken as the case study, and a hidden Markov model (HMM) was trained over its observed symptoms. The data used for the analysis was the outcome of a study hosted by Welltory. It involved the collection of SAR-Cov-2 symptoms and reading of body vitals using Apple Watch, Fitbit, and Garmin smart bands. The internal states of the HMM were made up of the absence and presence of a consistent decline in standard deviation of NN intervals (SSDN), the root mean square of the successive differences (rMSSD) in R-R intervals, and low frequency (LF), high frequency (HF), and very low frequency (VLF) components of the HRV measurements. The emission probabilities of the trained HMM instance confirmed that the onset or worsening of the symptoms had a higher probability if the HRV components displayed a consistent decline state. The results were further confirmed through the generation of probable hidden states sequences using the Viterbi algorithm. The ability to pre-empt the exigent state of an affliction would not only lower the chances of complications and mortality but may also help in curbing its spread through intelligence-backed decisions.

## 1. Introduction

The IoT-enabled products have made a foray into our daily lives in a multitude of forms. These forms could vary in size and mobility [1,2,3]. For instance, a smart kettle could be stationed on a kitchen counter and not take up much space. On the contrary, a smart grid management system would be more complex and large-scale. With constantly evolving semiconductor technology, it is possible to host IoT-based intelligence on an even smaller scale. Such advances have popularized smart devices that can be worn in the form of a fitness band, a smart garment implanted with sensors, or something as futuristic as a chip implanted into the skin like a tattoo [4]. Such devices, termed as the smart wearables, rely on perceptive sensors to monitor user location and activities and exchange this data with cloud-hosted services to assist in providing a better living experience for the user [5,6,7]. While these devices were introduced to bring fitness monitoring into the hands of the user; the development of advanced sensors, communication protocols, and data processing algorithms have made them into sophisticated tools for specific healthcare requirements. For instance, a fertility monitoring system was developed that used a machine learning algorithm over temperatures recorded by an in-ear thermometer to predict the ovulation period [8].

If we delve briefly into the history of smart wearables, the abacus rings that were popular during the Chinese Qing Dynasty era emerge as the early prototypes. Further centuries saw the first wearable timepiece that was introduced in the year 1505, or a two-piece computing device developed by two MIT professors to cheat on a game of roulette in the year 1961.Furthermore, the wristwatch containing a calculator marketed by Pulsar in the year 1975 [9]; shows that wearable computing has always stoked interest among the masses. The world of portable music players and basic wearable electronics was revolutionized by the invention of networking technologies like Bluetooth, NFC, RFID, and Wireless networks. The initial smart wearables had limited functionality, an example being early smartwatches that relied on the connected smartphones for sensing and operations. With the evolved supporting infrastructure, smart wearables graduated to headwear, footwear, eyewear, and other modular designs [10,11]. To date, wrist-worn smart wearables that are comprised of smartwatches and fitness bands still command the majority of sales [12].

The keen acceptance of wristbands among users belonging to different demographics could be attributed to their non-intrusive wearability and potential for aesthetics. Presently they are being used not just for location and activity monitoring, but in conjunction with dedicated mobile applications, they now offer support for specific health problems [13,14]. For instance, the wristbands like Brio, Pulse Companion, and Open Seizure Detector can detect epileptic seizures and summon help based on the wearer’s pulse activity [15].The capability offered by such wristbands and fitness trackers to record various body vitals while supporting unobtrusive long-term wear have opened exciting avenues to further healthcare research.

**Figure 1:**
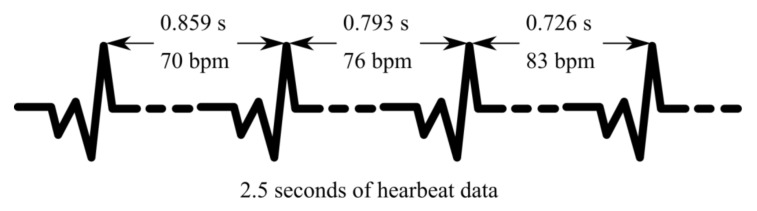
The representation of heart rate variability (HRV), where the peaks mark the heartbeats and the time between them. Source: www.heartmath.com/science/.

Among the vitals monitored by smart wristbands, heart rate variability (HRV) has emerged as an important parameter. HRV is defined as the variation in the time that elapses between consecutive heartbeats, measured in milliseconds [16,17]. Being a neuro-cardiac function, it is the result of interactions between the heart and the brain, thereby indicative of the body’s adaptability to environmental and psychological challenges [18,19,20]. Figure 1 shows how changes in heartbeats make up the HRV. The HRV of a person is governed by two aspects of the autonomic nervous system, namely sympathetic and parasympathetic branches [21]. The sympathetic part regulates the body’s reaction to mental and physical stress by the release of suitable hormones, thereby causing an increase in the heart contractions and reducing the HRV. Its counterpart, the parasympathetic branch regulates the body when it has to recover from a stressful state. To restore the body to a relaxed state, the parasympathetic branch slows down the heart rate and thus increases the HRV. Studies have shown that HRV can be used to predict morbidity caused by mental disorders like depression and post-traumatic stress syndrome, or physical afflictions like diabetes, concussion, asthma, and so on. The HRV is therefore a good indicator of underlying health issues and an important factor that can be used to lower the chances of mortality [22]. 

The significance of HRV measurements to pre-empt critical states of an illness or ineffectiveness of a treatment has been established by several researchers. The availability and cost of diagnostic tests often discourage people from seeking help until the symptoms of an illness become extreme, and at times the illness has advanced beyond the possibility of treatment. Therefore, a well-designed system that can interpret a person’s HRV measurements, detect possible underlying health issues, and alert the wearer to seek help, could prove to be a life-saving strategy. Moreover, the availability of healthcare facilities suffers greatly during the time of a pandemic, since they get overwhelmed by the volume of patients. It could also result in the late detection of critical stages of health conditions and increased mortality figures. In the year 2020, the pandemic of SARS-Cov-2, commonly known as Covid-19, had afflicted millions of people over the world and across all demographics. Its large- scale spread could be mostly attributed to inadequate tests, late detection, and overwhelmed healthcare systems [23,24]. In this paper, we hypothesized that the observations of HRV readings can be used to pre-empt the onset and criticality of a pandemic illness, and consequently stem and control its spread. Due to its relevance to current times, we have taken Covid-19 as a use-case to back the hypothesis. We analyzed HRV records of participants of Welltory Covid-19 research [25], who logged their Covid-19 symptoms and shared the various vitals that were collected by commonly-used fitness monitoring mobile applications. A hidden Markov model-based framework was developed to discern how trends followed by HRV components were associated with the progression of Covid-19 affliction.

In the Section 2, we discuss the use of smart wearables and the applicability of HRV measurements in context with healthcare. This is followed by the description of the data that supports the case study, and the principle and architecture of the method used for the analysis. We show the results achieved through the analysis, and discuss the outcome of the hypothesis and potential for future use in Section 3, Section 4 and Section 5.

## 2. Background

In this section, we delve into the research that has been carried out in the field of smart wearables in the biomedical and healthcare domains. Since we focused on HRV as an important factor among the body vitals, we also studied research done over its applicability to health monitoring. Lastly, we summarize our understanding of the existing research by listing the identified gap areas that motivated us to develop the proposed framework and contribute to this area of research.

### 2.1. Smart Wearables in the Healthcare Domain

Smart wearables that record body vitals such as heart pulse temperature and the state of the wearer’s skin were developed in recent years. Some relevant examples include a chest belt that monitored body temperature, heart, and breathing rates to alert against Sudden Infant Death Syndrome [26]. Another innovation involved a Micro-Electro-Mechanical Systems (MEMS) technology integrated with a mobile application to protect against ankle sprains [27]. Ankle sprains were reported as the most commonly reported sports injury. Another wearable sensor worn on the skin could detect hydration levels and alert against complications that could arise in diabetic persons [28]. For serious afflictions like heart ailments, a touch device was developed to track bio-parameters like an electrocardiogram; it tracked SpO_2_, skin temperature, and the physical activity of the patient [12]. This device provided a means for the patient to keep track of their vitals from home. Smart wearables could also be used to aid patients of Parkinson’s disease [29]. Hand tremors are one of the debilitating effects of Parkinson’s that cause interruption in the daily life of the patient. A low-cost assistive wearable was developed to inhibit the bio-mechanical feedback loop between the brain and the hand to reduce the hand tremors and thereby improve the patient’s gripping capability.

The advent of fitness trackers and wrist-worn smart devices opened further avenues for biomedical research. The user data could be recorded over longer periods and supporting mobile applications provided means to log relevant user information. Early comparison of heart rate measurability between the Apple watch and the Polar chest strap showed that under normal conditions, their performance was commensurate [30]. The wrist-worn monitors like Apple Watch use a combination of LED lights and sensitive photo-diodes as a part of the PhotoPlethysmoGraphy (PPG) process. An optimum combination of LED brightness and sampling rate can aid in effective sensing of the heart rate. Another device worn on a subject’s forearm could help in the rehabilitation of patients after stroke [31]. The smart wearable consisted of an armband containing electrodes. Its functions were powered by machine learning algorithms, and a robotic hand that could mimic the purported actions of the patient. A life-saving wrist wearable was also developed to monitor pulse and EMG signals in a non-hospital environment [32]. This device was proposed as a rapid response tool to prevent sudden deaths before the wearer could reach the hospital. Arm-worn smart devices can also be used to monitor arm movements that are an important feature of the walking gait of humans [33]. An analysis of the arm swinging motion can help in the detection of motor dysfunctions such as those found among patients of Parkinson’s disease.

### 2.2. Heart Rate Variability as a Health Indicator

In Section 1, HRV was introduced as the fluctuations in the time interval that elapses between consecutive heartbeats. The spatial and temporal properties of a person can be mapped to HRV measurements based on two types of metrics [15]. The first type of metric is recorded in the time domain and quantifies the HRV behavior during the monitoring periods that may span from one minute to twenty-four hours. The important time-domain HRV metrics include the standard deviation of NN (SDNN) intervals, the standard deviation of the average R-R intervals (SDANN), the root mean square of the successive differences (RMSSD) in R-R intervals, and the percentage of normal R-R intervals that differ by 50 ms (pNN50). The SDNN is considered as the “gold standard” for the prediction of both morbidity and mortality in case of a cardiac risk [16,17,34]. Patients, monitored over 24 h, are deemed unhealthy if their SDNN values are found to be below 50ms.HRV metrics in the frequency domain can be obtained using fast Fourier transformation or autoregressive modeling. This way, the rhythms in ULF, VLF, LF, and HLF regions can be separated into separate components. According to medical research, the LF/HF ratio serves as an index to assess the relationship between the sympathetic and parasympathetic processes and therefore mark the variation in type and handling of causal stress [34]. An interesting application for drowsiness detection was developed that relied on HRV signals to discern dominant respiratory characteristics [35].

### 2.3. Covid-19 and Cardiovascular Health

Due to its relevance with current times, the proposed framework was implemented with the Covid-19 pandemic as the use-case. The first known instance of human SARS-Cov-2 infection was recorded in the year 2002. A cohort study on 121 patients confirmed that Corona virus infection manifested itself in the form of hypotension, cardiac arrhythmias, and even sudden cardiac death [36,37]. With the SARS-Cov-2 infection taking on pandemic proportions at the end of the year 2019, renewed research was carried out to reveal the underlying trends. Extensive clinical research on patients determined that while SARS-Cov-2 caused myocardial injuries, pre-existing cardiovascular issues in patients aggravated the extent and chances of mortality [38]. On one hand, preventive measures like social distancing and hygiene practices were promoted widely, on the other, research was accelerated for its early detection. Jiangsu province identified four risk factors of age, lymphocyte count, oxygen supplementation, and aggressive pulmonary radiographic infiltrations, to develop an early warning system and determine high-risk patients [39]. This strategy resulted in the effective curbing of the spread despite high initial transmission figures. An Arduino-powered wearable system was also proposed to monitor vital signs like body temperature, heart rate and respiration rates to warn the wearer and remote medical service providers about the possibility of a SARS-Cov-2 affliction [40].

### 2.4. Motivation and Contribution

The vast application prospects offered by smart wearables led us to explore further utilization of avenues in the healthcare domain. We also studied how the HRV measurements had found acceptance as a critical informative factor for pre-emption of mental and physical health issues. Yet it had not been applied for pre-emption of onset and criticality during a pandemic situation. The gap area was more apparent since the year 2020 had seen the outbreak of SARS-Cov-2 on a worldwide scale. To our best knowledge, this was the first time that a machine learning-based framework was designed to predict the onset and increased severity of an infection such as SARS-Cov-2. It was also to our best knowledge that the HRV measurements had been used by an algorithm to explore its applicability in pre-emptive diagnostics. We propose a hidden Markov model (HMM) based framework, driven by SARS-Cov-2 symptoms that were logged by the participants of a research study hosted by Welltory. The hidden states of the aforementioned HMM comprise changes in the standard deviation of NN (SSDN), rMSSD, and the low frequency (LF), high frequency (HF), and very low frequency (VLF) components of the HRV measurements that the participants had recorded during the course of the study. We hypothesized and proved that the onset and exigent state of SARS-Cov-2 affliction can be predicted to a certain degree through the analysis of variations in the HRV components.

## 3. Materials and Methods

### 3.1. Data Acquisition

The framework proposed in this paper was based on the principle of monitoring the trends followed by the use of HRV time and frequency based metrics to predict the onset and oncoming exigent state of a major illness. We had specifically focused on SSDN, rMSSD, LF, HF, and VLF for the same. To test our hypothesis we modeled the framework using data published by Welltory [41]. 

Welltory invited participants who had been afflicted with SARS-Cov-2 virus to share the progress of their symptoms, and body vitals including the HRV collected by wearables like Apple Watch, Garmin, and Fitbit in collaboration with the Welltory mobile application. The data was collected over the early months of the year 2020 when the SARS-Cov-2 pandemic had started to spread worldwide. Due to the new laws that forbade the release of mobile applications with titles containing Covid-related terms, the Welltory application had to be removed from the app marketplaces, thus ending the data collection. Participants had shared HRV measurements through PPG technology, while other vitals were collected through wrist-worn smartwatches and bands that were synchronized with the Welltory app. The data that was published by Welltory for researchers was anonymized and was comprised of multiple CSV files.

### 3.2. Process Design

#### 3.2.1. The Proposed Architecture

**Figure 2:**
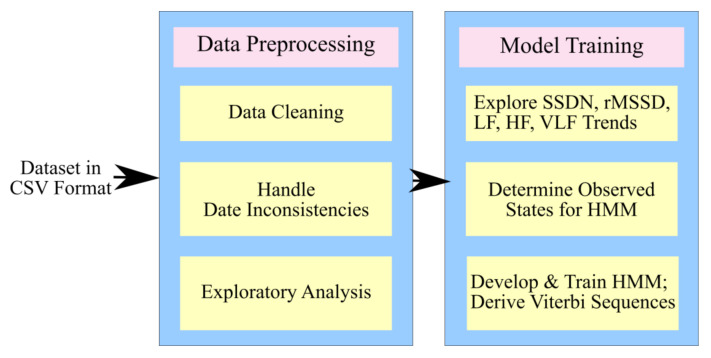
The architecture of the proposed framework.

The proposed framework was visualized with two major functionalities. As can be seen in Figure 2, the first component was responsible for the data pre-processing functionality. Since the data was acquired from Welltory Labs, it needed relevant cleaning and exploration. The date fields were significant factors in the study, and their inconsistencies and absence were handled as part of pre-processing. The dataset was found to contain participants who spanned across countries and age groups, and had proportionate gender-wise figures. It was also found that the reporting behavior of the participants was not uniform. While some participants had reported symptoms and HRV measurements diligently, many other participants did not log SARS-Cov-2 symptoms for more than a few days. The absence of cases where participants had recovered and contracted SARS-Cov-2 again also left opportunities for future research.

The second component of the proposed architecture was responsible for the conversion of reported SARS-Cov-2 symptoms into a sequence of observations. It was also responsible for the monitoring of trends followed by HRV components to discern the hidden states. The observations and hidden states were then used to train an HMM–based system and derive relevant probabilities. Figure 2 shows the simple depiction of the proposed architecture. The HMM details and relevant logic flow are discussed in the Section 3.2.2.

#### 3.2.2. Hidden Markov Model for HRV Trends and SARS-Cov-2 Symptoms

The hidden Markov model is a sub-case of Bayesian networks and a generative probabilistic model. It is characterized by a set of internal hidden states S = {s_1_, s_2_,…, s_N_} and a generated sequence of observations X = {x_1_, x_2_,…, x_N_} [42,43]. While the internal states are not observed explicitly, they are assumed to display first-order Markov chain form, which means that the probability of transitioning to a state is solely dependent on the current state and the time parameter. The mathematical form of an HMM can be written as the following equation:

Θ = {Π, T, E},(1)

where Π_i_ = P(x_1_ = s_i_) are the prior probabilities of s_i_ being the first state of a sequence and x_1_ the first observation. The matrix T is the collection of probabilities of transitions between two states i and j. Each element of the matrix T can be written as:

t_i,j_ = P(x_n+1_ = s_j_|x_n_ = s_i_), (2)

And lastly vector E(x) of functions summarizes the emission probabilities, that is, the likelihood of the observation x if the HMM were in state s_i_.

The three fundamental scenarios that can be addressed by an HMM are first, the estimation of the optimal sequence of hidden states if the model parameters and observations had been provided. Secondly, if the model parameters and observations had been provided, then calculate the model likelihood. And lastly, estimate the model parameters if only the observations had been provided. The first two scenarios are use-cases for the application of dynamic programming algorithms such as the Viterbi and the forward-backward algorithms. The last scenario can be solved by the Baum-Welch algorithm [44] that is based on the expectation-maximization principle.

**Figure 3:**
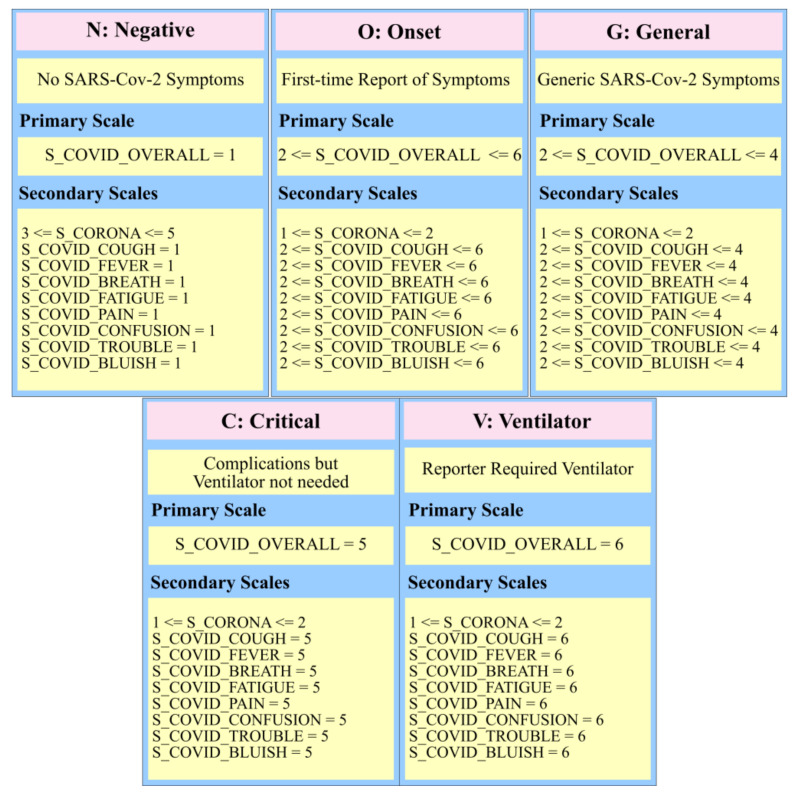
The reported symptoms and severities mapped to hidden Markov model (HMM) observations.

The rationale behind the application of HMM in the proposed framework was that the SARS-Cov-2 symptoms recorded by the participants followed a logical sequence. Moreover, the variations in the five HRV components, namely SDNN, rMSSD, LF, HF, and VLF, were hypothesized to have a direct impact on the observed symptoms and could be used to predict the progression of SARS-Cov-2. The hidden states for HMM were thus labeled as 1 and 2, where label 1 was for the case when no continuous decline in the HRV measurements was noticed. The label 2 was used to convey the hidden state when the five relevant HRV components had dropped over a span of a week. For this study, the various symptoms that were logged by the participants were categorized into specific labels as shown in Figure 3. These labels were used as observations to train the HMM. It should be noted that a participant could transition among the observations based on the progression of the SARS-Cov-2 affliction. For instance, a participant could have reported the onset of the Covid-19 affliction and sought treatment. But due to inherent health conditions, the treatment was not effective and the participant progressed to a critical state. The participant, therefore, logged a sequence of “NOVV” for their observations, instead of “NOGVV”. We refrained from interfering with the participant logging behavior and translated the recorded severity truly per the participant’s input rather than according to an expected sequence.

A hidden Markov model is characterized by the combination of probabilities for a set of hidden states and discrete observations. The Baum-Welch algorithm determines the expected maximum likelihood of parameters of an HMM if given a set of observations. It uses the expectation-maximization principle since the calculation of all possible transitions between the postulated states would result in a large-scale computation problem. The Viterbi algorithm is a dynamic programming technique to determine an optimal sequence of hidden states for a given HMM and a set of observations [45]. Firstly the Baum-Welch algorithm was used for every individual sequence of observations reported by the participants. The algorithm accepted the observation sequences and the two hidden states to generate emission and transmission probabilities. The Viterbi algorithm was then used on the trained HMM instance and some sample observations to generate an optimal sequence of transitions between the hidden states. These hidden state transition sequences were checked against the previously detected states for the sample observations to test HMM performance.

### 3.3. Preprocessing and Exploratory Analysis

**Figure 4:**
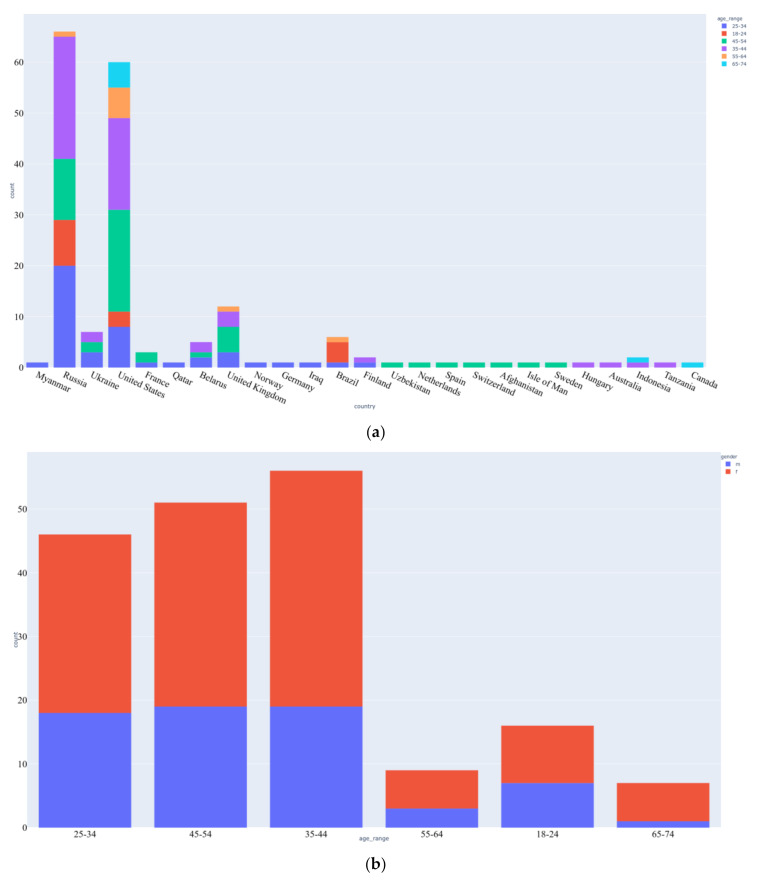
The distribution of the participants over their (**a**) age groups and countries of residence and (**b**) gender and age group.

The first CSV file contained anonymized information of the 186 participants, specifying their gender, age group, country of residence, and the date when they observed the onset of SARS-Cov-2 symptoms. The dataset had many missing values for the onset date, while a few participants had instead put their birth dates in that field. Figure 4 shows the age, gender, and country-wise distribution of the participants that we derived from the provided data.

**Figure 5:**
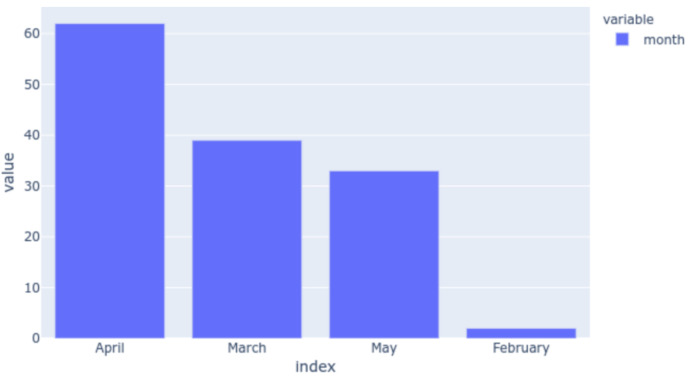
The distribution of the onset months reported by the participants during the study.

The data collection through the Welltory app had taken place from February 2020 until May 2020 and the distribution of the onset months that we plotted is shown in Figure 5. During the course of the study, the month of April saw the reporting of the maximum number of SARS-Cov-2 symptoms.

In a separate CSV file, the SARS-Cov-2 symptoms and the pre-existing health conditions that had been shared by the participants were listed, along with the observed severity, ranging in value from 1 to 6. The SARS-Cov-2 symptoms severities specified absence with value 1, very mild severity as 2, mild severity as value 3, value 4 as moderate, value 5 as severe and value 6 was used to indicate extremely severe symptoms. The symptoms for SARS-Cov-2 were labeled under different scales as shown in Table 1.

**Figure 6:**
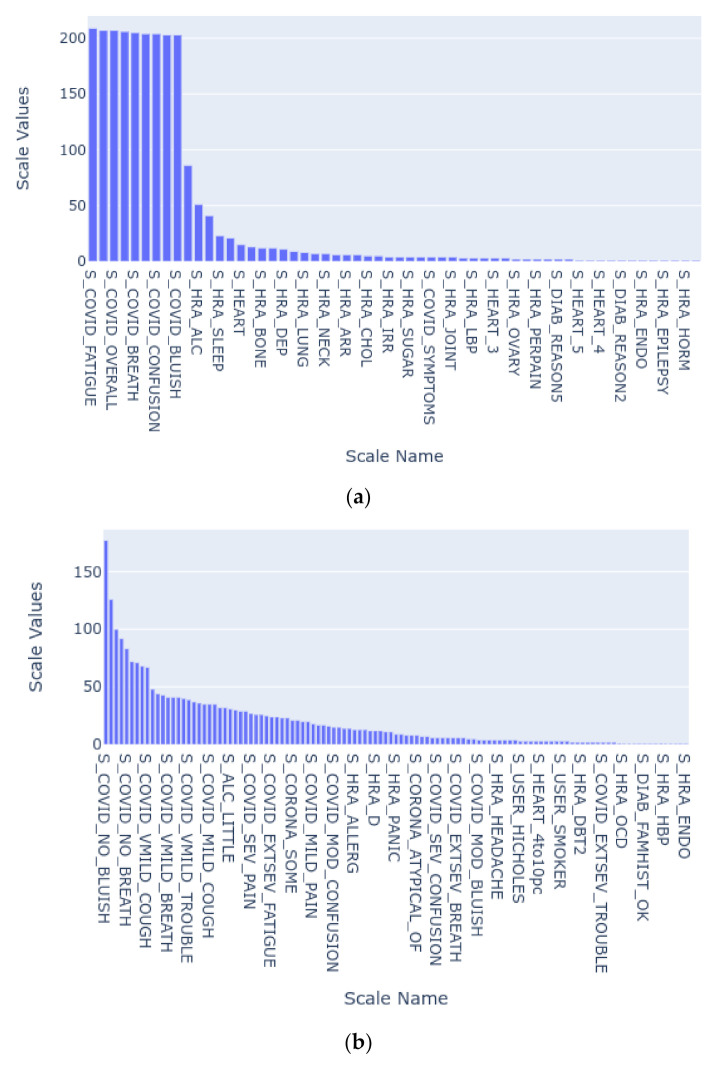
The distribution of the SARS-Cov-2 and pre-existing conditions logged by the participants: (**a**) irrespective of the severity and (**b**) with severity.

The participants also logged pre-existing conditions that specified if the person was suffering from heart conditions, high blood pressure, diabetes, arrhythmia, thyroid problems, and so on. The participants also recorded mental issues such as anxiety, insomnia, and the post-traumatic syndrome. We plotted the distribution of conditions that were highly reported by the participants, irrespective of their severity. We then collapsed the conditions over the severity and plotted their distribution. The two graphs are shown in Figure 6.

As can be seen in Figure 4, SARS-Cov-2 related symptoms were the most reported conditions in the dataset. On analysis based on the reported severity, it can be seen that majority of participants experienced moderate severity of SARS-Cov-2. While negligible shortness of breath and no bluishness of skin or lips were reported, severe to mild chest pain was experienced by the reporters. A small number of reporters had also experienced extreme confusion and extreme shortness of breath. Among the non-SARS-Cov-2 conditions, little alcohol consumption, allergies, vitamin D deficiency, panic, and smoking habits were observed to be present in the majority of participants.

**Figure 7:**
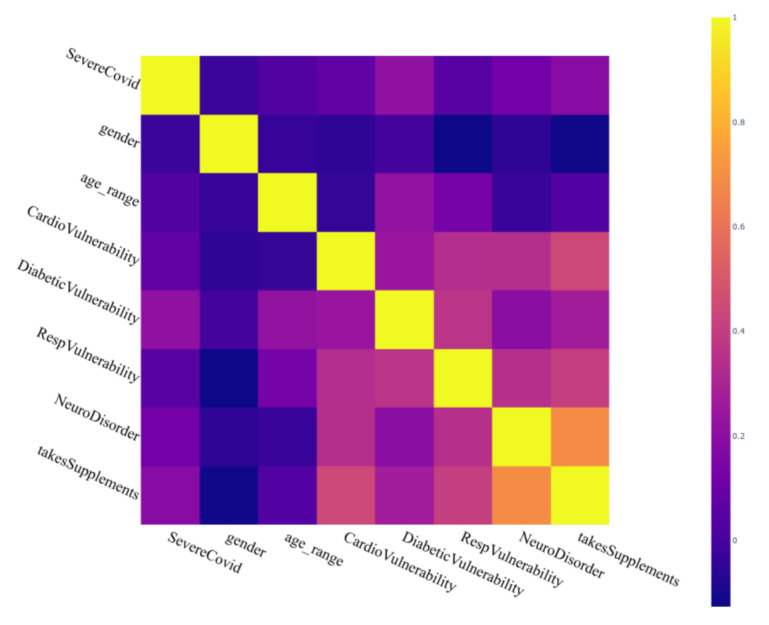
The correlation observed between some major health conditions and the overall SARS-Cov-2 state reported by the participants.

To achieve a better understanding of reported conditions and SARS-Cov-2 symptoms severity, the correlation was plotted between gender, age, cardiovascular vulnerability, diabetic conditions, respiratory vulnerability, the presence of neurological disorders, participant partaking of vitamin supplements regularly, and participants having reported a severe S_COVID_OVERALL symptom. From Figure 7, we can see that while participants who had reported neurological disorders had a high probability of taking supplements, both of the conditions had a low positive correlation with severe S_COVID_OVERALL symptom. Due to a low number of elderly participants, as confirmed in Figure 4b, we could not derive much of the relationship between the conditions that are typical of old age and the probability of getting afflicted by SARS-Cov-2. It should also be noted that the participants who had recorded respiratory or cardiovascular vulnerabilities had a positive correlation with severe SARS-Cov-2 affliction.

### 3.4. The SARS-Cov-2 Detection Prototype

**Figure 8:**
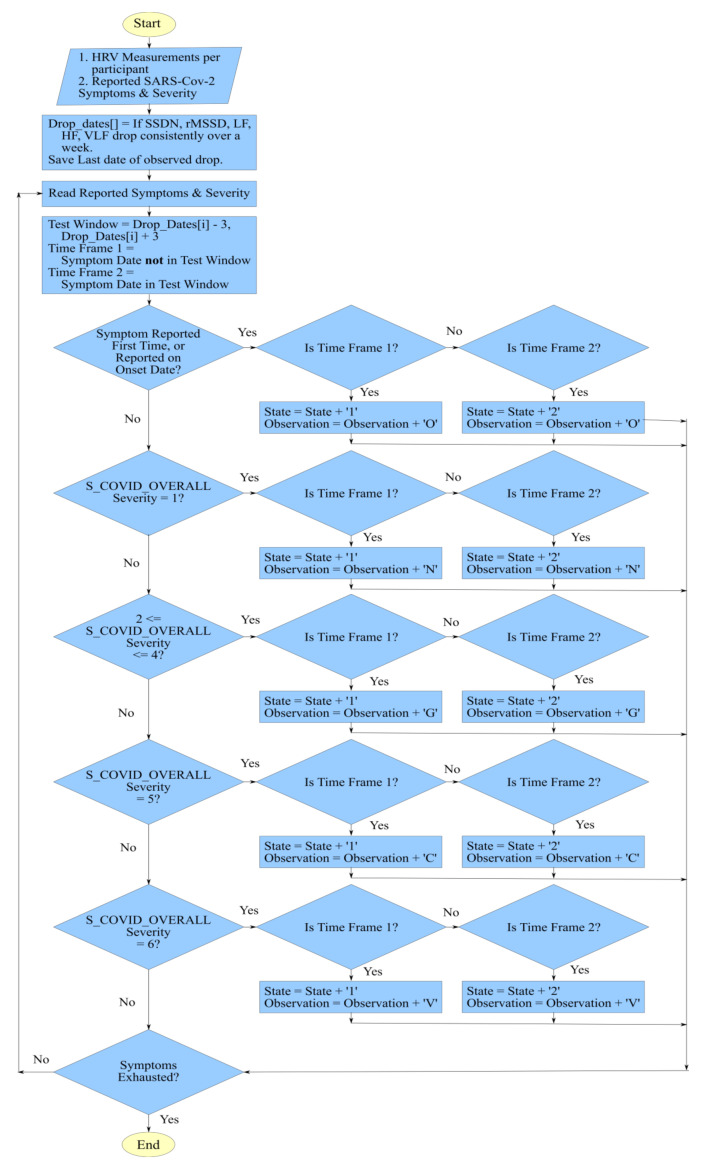
The flow of logic used to generate observations and hidden state sequences for HMM.

The proposed framework was implemented using a script written in Python programming language version 3.8.5 on a system running on Linux Mint OS 20 Ulyana using the JupyterLab IDE. The first part of the script pre-processed the CSV format files that were received in the dataset. The data was further explored and relevant visualizations were plotted using the Pandas and Plotly Express libraries. The subsequent part of the script focused on the extraction of observations reported by the participants and the hidden states computed using the behavior of the five HRV components. Figure 8 shows the logic that was used for this functionality. To simplify the flow diagram, we only focused on various severities of symptom S_COVID_OVERALL, while the executed script also considered the secondary SARS-Cov-2 symptom scales.

To discern the hidden states, drops in the values of the five HRV components were monitored. If a consistent decline was seen over a week for all of the five components, then the last date of the time window was saved. The algorithm saved dates for every consistent decline that spanned over a week for complete HRV measurement data shared by each participant. The significance of drop dates in the determination of hidden states was based on the theory that a symptom observation made in a time window far from a drop date would negate the hypothesis. Therefore, we considered two timeframes; the first frame covered a six-day window with three days prior and after a drop date. The second time frame covered the dates that did not lie in a relevant drop window. By traversing through the reported SARS-Cov-2 symptoms and their severity, we built a sequence of hidden states and observations. The hidden states could either have a value of 1 or 2 depending on the reporting of the SARS-Cov-2 symptom outside or within a drop window, respectively. The observations could be any of the labels that were introduced in Section 3.2.2, namely N, O, G, C, and V. The generated sequences were used to train an HMM instance according to the Baum-Welch algorithm. The HMM class was imported from a third-party library available for public use [46].

The trained HMM instance was plotted to see the transition and emission probabilities with the help of the Graphviz library [47]. Also, observation sequences of six randomly selected participants were used to generate the hidden state sequences using the Viterbi algorithm. The actual and generated sequences of hidden states were then compared to discern the performance of the HMM instance.

## 4. Results

**Figure 9:**
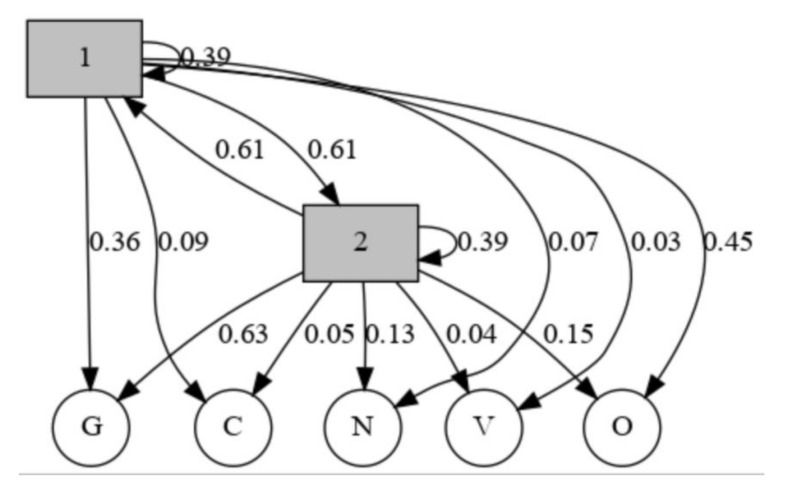
The emission and transition probabilities of the developed hidden Markov model. The hidden states 1 and 2 mark the absence and presence of a consistent decline in five major HRV components, respectively. The observations G, C, N, V, and O mark the SARS-Cov-2 symptoms reported by the participants.

The proposed framework for pre-emption of onset and criticality of infection was implemented using SARS-Cov-2 data as a use-case. The behavior followed by five major HRV components were monitored for a consistent decline and thus marked for further use. An HMM instance is made up of two important sequences, the observations and the hidden states. The observed states of the developed HMM instance were achieved through the symptoms logged by the participants. Figure 9 shows the graphical representation of the developed hidden Markov model along with the relevant probabilities of emission and transition. The probabilities were calculated using the Baum-Welch method.

The transition probabilities between the two hidden states are shown in Table 2.

The emission probabilities for both the hidden states and the five observations are shown in Table 3.

As can be discerned from Figure 9 and Table 2 and Table 3, the emission probabilities from state 1 to any of the five observations are lower than the emission probabilities associated with state 2. Therefore, if a person was not displaying a consistent decline in the five major components of HRV, or in other words if the current hidden state was 1, then their probability of reporting Generic, Critical, or Severely Critical symptoms would be lower than when in state 2.

**Figure 10:**
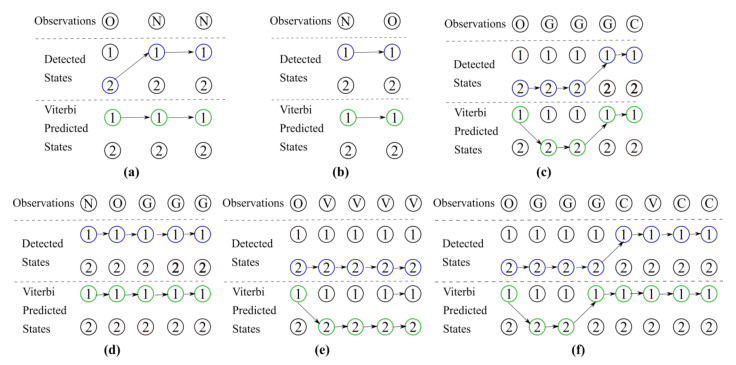
The trellis diagrams to compare sequences of hidden states as discerned from the HRV measurements, and those predicted by the Viterbi algorithm. (**a**) The reporting participant did not experience any symptoms after reporting the onset of SARS-Cov-2. (**b**) The participant had logged SARS-Cov-2 symptoms in accordance with an expected order of going from Onset to Generic symptoms observation. (**c**) The participants SARS-Cov-2 affliction had become Critical. (**d**) This participant had also logged symptoms observations that were in an expected order of Negative to Onset, and then to Generic symptoms. (**e**) The participant had logged the Onset and next record was made when the affliction had become Critical. (**f**) The longest observed sequence that also covered four different states of Onset, Generic, Critical and Ventilator support of SARS-Cov-2 affliction.

To further test the performance of the trained HMM instance, observations of six randomly selected participants were used as input to the Viterbi algorithm. The Viterbi algorithm gave the probable sequence of hidden states derived from the input observations. The selected hidden state sequences that were used to train the HMM were thus compared against the Viterbi-generated state sequences. Figure 10 shows the trellis diagrams of the training and generated hidden state sequences. The randomly selected sequences varied in lengths and progression and therefore tested the accuracy of the model for different reported scenarios. It can be ascertained that the transitions between two hidden states for the participants were similar to those generated using the Viterbi algorithm.

Figure 10 shows the comparison of hidden state sequences determined through our scripts, against the state transitions calculated by the Viterbi algorithm, for given input observations. The input observations varied over lengths and SARS-Cov-2 progression, and thus ensured a comprehensive test set. The test observation Figure 10a showed a negative progression with the reporter experiencing no symptoms after reporting the onset of the infection. The detected and Viterbi-predicted hidden state transitions differ for only one state. While progression samples Figure 10b,d that followed an expected order of going from Negative to Onset in Figure 10b, and Negative to Onset to Generic symptoms in Figure 10d, the hidden state sequences match. For the samples, Figure 10c,e when reporters had experienced critical symptoms, the hidden state sequences again differ slightly. It can be observed that the detected and Viterbi-predicted hidden state transitions agree. Lastly, a long sample observation sequence Figure 10f was used to test the performance of trained HMM to uncover hidden state transitions over four different observations of O, G, C and V. For a sequence of length 8, only two hidden states were found to differ.

## 5. Conclusions

The advent of IoT in healthcare has created a playing field for researchers and developers to find innovative solutions for timeworn problems. The smart wearables, especially the wrist-worn technology not only appeals to aesthetics but also facilitate long-term wear, thus providing scope for extensive data collection. The human body is a machine that is governed by regulated processes, and disruptions in such working processes can be attributed to mental or physical stress. The evolving sensitivity of the smart wrist-worn device has therefore found extensive use in monitoring of body vitals and provides data for algorithms to discern any health issues that might afflict the wearer. We proposed a hidden Markov model-based framework that could be trained over variations of five major HRV components, namely SSDN, rMSSD, LF, HF, and VLF, to alert against onset or worsening of an affliction. Due to its significance at the time of the study, SARS-Cov-2 was taken as a case study and the HMM was trained over SARS-Cov-2 symptoms recorded by participants of research hosted by Welltory.

We hypothesized that a consistent decline in the values of major HRV components could be attributed to the onset or worsening of SARS-Cov-2 infection. The probabilities reported by the modeled HMM instance proved that the emission probabilities between the hidden state of consistent decline and exigent SARS-Cov-2 observations were higher when the participant had experienced consistent drops in the five HRV components. The Viterbi algorithm was used to generate sequences of transitions between the hidden states, and the outputs were found to agree with the transitions detected from the HRV readings. The data collection process was dependent on the logging behavior of the participants; therefore, we followed the actual reported observations rather than according to the expected progression of SARS-Cov-2 infection. Despite the errant and limited observations in some cases, the developed HMM model worked well. Moreover, since the participant data could be collected for only three months, there is a vast scope for further investigations. A larger dataset would provide more variant observations and better testing strategies.

The proposed framework showed that HRV measurements taken by wrist-worn smart wearables have a vast potential for future research. It also showed that such data collection and observations recorded by people with limited technical knowledge did not affect the performance of the developed system. For future work, we would test the framework for other afflictions and larger datasets. We are also hopeful that this study will encourage further research in the usability of data collected by wrist-worn fitness bands to pre-empt afflictions before symptoms appear, and thus lower the chance of complications and mortality.

## Figures and Tables

**Table 1:** The scales used by the participants to quantify their SARS-Cov-2 symptoms.

Scale Name	Description
S_COVID_OVERALL	Overall State
S_COVID_SYMPTOMS	How long the user had been experiencing the symptoms
S_COVID_COUGH	The intensity of coughing
S_COVID_FEVER	The intensity of fever
S_COVID_BREATH	The intensity of shortness of breath
S_COVID_FATIGUE	The intensity of fatigue
S_COVID_PAIN	The intensity of pain or pressure in the chest
S_COVID_CONFUSION	The intensity of confusion
S_COVID_TROUBLE	The intensity of trouble in breathing
S_COVID_BLUISH	The intensity of bluish face or lips
S_CORONA	An assessment of reported symptoms to show how likely the reporter has Covid-19

**Table 2:** The transition probabilities of the two hidden states, where value 1 maps to the HMM hidden state when the participant HRV components did not show any consistent decline, and value 2 maps to the HMM hidden state when the participant HRV measurements showed a consistent decline.

	1	2
**1**	0.39021	0.61178
**2**	0.61178	0.39021

**Table 3:** The emission probabilities between the two hidden states, 1 and 2, and the five observations, N, O, G, C, V.

	N	O	G	C	V
**1**	0.065553	0.45182	0.362460	0.093164	0.027004
**2**	0.131894	0.14845	0.633617	0.050245	0.035794
